# Numerical analysis of MICP treated sand based on bio-chemo-hydro model

**DOI:** 10.3389/fbioe.2024.1380213

**Published:** 2024-03-22

**Authors:** Delong Li, Shengzhe Chen, Xing Gao

**Affiliations:** Yantai Yuhuangding Hospital, Yantai, China

**Keywords:** bio-grouting, microbially induced calcite precipitation (MICP), soil improvement, bio-chemo-hydro model, numerical method

## Abstract

Microbially Induced Calcite Precipitation (MICP) represents an environmentally friendly and innovative soil grouting technology. Involving intricate biochemical processes, it poses challenges for a thorough investigation of factors influencing microbial grouting effectiveness through experimentation alone. Consequently, A three-dimensional numerical model was developed to predict the permeability of bio-grouting in porous media. The numerical model is validated by comparing its results with test results available in the literature. The validated model is then used to investigate the effects of variation bacterial solution concentration, cementation solution concentration, grouting rate and grouting time on grouting effectiveness. It was founded that the remediation effect was positively correlated with the bacterial solution concentration and the number of grouting. An increased grouting rate enhanced the transport efficiency of reactants. Additionally, the concentration of cementation solution exhibited no significant effect on the reduction of calcium carbonate yield and permeability.

## 1 Introduction

Microbially induced calcite precipitation (MICP) is a green and innovative soil grouting technology (Mitchell and Santmarina, 2005; Dejong et al., 2006; Van Paassen et al., 2010; Wu et al., 2021; Murugan et al., 2022). The technology can effectively improve the mechanical and hydraulic properties of porous materials such as sands and soils by using the filling and cementing effects of the mineralization product calcium carbonate (Ivanov and Chu, 2008; Soon et al., 2013; Chen et al., 2023). Numerous studies have demonstrated that MICP technology shows potential for application in several research fields, such as soil reinforcement, fissure repair, and bio-inspired heavy metal immobilization (Wang K. et al., 2023; Xie et al., 2023; Xue et al., 2023). Meanwhile, It has a wide range of applications in the field of geotechnical engineering (Van et al., 2010; Chu et al., 2012; Choi et al., 2016; Jiang and Soga, 2017; Xiao et al., 2019; Wu et al., 2019; Wang and Nackenhorst, 2020; Liu et al., 2023).

Previous experimental studies have found that the mechanical and hydraulic properties of the material after microbial grouting reinforcement are closely related to the production and distribution of the mineralization product calcium carbonate (Al Qabany et al., 2012; Tobler et al., 2012; Zeng et al., 2019; Li et al., 2023; Tang et al., 2023). However, there are numerous factors affecting the microbial mineralization products, including bacterial solution concentration, cementation solution concentration, calcium source type, temperature, ambient pH, grouting rate, and grouting frequency, etc (Mortensen et al., 2011; De Muynck et al., 2013; Soon et al., 2014; Amarakoon and Kawasaki, 2016; Rowshanbakht et al., 2016; Al Salloum et al., 2017; Keykha et al., 2017). In addition, some researches have shown that the samples treated with higher solution concentrations showed the formation of precipitates on the surface of the sand. Clogging formation is a common problem during the biocement treatment when using percolation or injection treatment (Dhmai et al., 2013; Cheng and Cord-Ruwisch, 2014). In the past decade or so, many researchers have conducted experimental studies on MICP, gaining a systematic and reasonable understanding of the processes involved (Wang L. et al., 2023; Fu et al., 2023).

Besides carrying out experiments, numerical simulation is also one of the methods for studying MICP technology (Ebigbo et al., 2012; Cuthbert et al., 2013; Qin and Hassanizadeh, 2015; Noiriel et al., 2016). The technology involves intricate biological, chemical, and percolation physical domains, including urea hydrolysis kinetics, multicomponent and bacterial transport, bacterial biofilm growth, equilibrium and kinetic geochemical reactions, calcium carbonate precipitation and porous media evolution (Fauriel and Laloui, 2012; Qin et al., 2016). Due to the complexity of this process, it is necessary to further establish a comprehensive bio-chemo-hydro model to accurately predict the repair effect of MICP technology. Van Noorden et al. (2010) deduced an effective model for biofilm growth in porous media and its impact on fluid flow, and considered the changes in pore volume caused by biomass accumulation in the model. Kim and Fogler (2000) used a porous micromodel to study the impact of biomass evolution on the permeability of porous media, and used a network model to describe the phenomenon of biofilm formation and the existence of critical shear stress. The simulation results are consistent with the experimental results, and the existence of critical shear stress is proved. Fauriel and Laloui (2012) established a general mathematical model to describe the injection, distribution, and reaction process of biological grouting in saturated, deformable porous media. The effects of adsorption and reaction on the composition of the solid matrix were also considered. The numerical simulation and theoretical analysis, have facilitated a more profound comprehension of the mechanisms and reaction kinetics underlying the biological-chemical process in recent years. However, the coupling mechanisms of multiple factors remains an issue in MICP. And in previous works, most of models were 1-dimensional or 2-dimensional, which fall to effectively reveal the spatiotemporal effects of various phases in MICP.

In this paper, a 3-dimensional numerical simulation method is used to describe the injection, distribution, and reaction processes of biological grouting in porous media systems. Subsequently, the effects of various factors on the effectiveness of microbial grouting is investigated through numerical simulation. These factors include bacterial solution concentration, cementation solution concentration, grouting rate and number of grouting. The numerical simulation results are analyzed to further deepen the recognition of the dynamic process of microbial grouting and to optimize the experimental scheme. Compared to extensive experiments, the effects of various factors on the effectiveness of bio-grouting be explored with relative ease by numerical simulation. This constitutes a significant advantage in situations where experimental resources are constrained.

## 2 Materials and methods

### 2.1 Methods

The chemical reaction process of calcium carbonate precipitation induced by microorganisms be summarized in two steps: (1) Urea hydrolysis, *Bacillus* pasteurei release a large amount of urease. Under the catalysis of urease, urea will hydrolyze to produce CO_3_^2-^ and NH_4_^+^. (2) The CO_3_^2-^ generated by urea hydrolysis reaction combines with the Ca^2+^ in the medium environment to form calcium carbonate precipitation. The specific reaction equation are as follows:

Hydrolysis of urea catalyzed by urease:

$$\text{CO}{\left({\text{NH}}_{2}\right)}_{2}\;\left(\text{aq}\right)+2H_{2}O\;\left(l\right)\;\overset{\text{urease}}{\to }\;2{\text{NH}}_{4}^{+}\;\left(\text{aq}\right)+{\text{CO}}_{3}^{2-}\;\left(\text{aq}\right)$$ (1)

Calcium carbonate precipitate:

$${\text{CO}}_{3}^{2-}\;\left(\text{aq}\right)+{\text{Ca}}^{2+}\;\left(\text{aq}\right)\;\to \;{\text{CaCO}}_{3}\;\left(s\right)$$ (2)

Combining the two steps involved in the above reactions (1) and (2), the total reaction equation for the MICP process can be obtained, as shown in Eq. 3:

$$\text{CO}{\left({\text{NH}}_{2}\right)}_{2}\;\left(\text{aq}\right)+2H_{2}O\;\left(l\right)+{\text{Ca}}^{2+}\;\left(\text{aq}\right)\;\overset{\text{urease}}{\to }\;{\text{CaCO}}_{3}\;\left(s\right)+2{\text{NH}}_{4}^{+}\;\left(\text{aq}\right)$$ (3)

The whole reaction process takes place in a porous medium environment. In the presence of urease, the chemical reaction catalyzed by bacteria leads to a decrease in the concentration of urea and calcium, and the rate of ammonium production depends on the bacterial concentration, urease activity and urea concentration. During microbial solidification, the precipitated calcium carbonate adheres to the sand particles, playing a filling and cementing role, reducing the porosity and permeability of the soil. At the same time, it also improves the strength and hardness of the soil.

To gain an in-depth understanding of the reinforcement and repair mechanisms inherent in MICP technology, this study undertakes simulations and analyses of the four influential factors: initial bacterial concentration, cementation concentration, grouting rate, and grouting time. Additionally, COMSOL finite element software is employed to model the three physical fields encompassing biology, chemistry, and hydraulics. The first is the biological field. The concentration changes and space-time distribution of bacteria in the porous media system will affect the rate of chemical reaction. The growth, adsorption and decay of bacteria are considered in the model. Chemical field refers to the consumption and diffusion of reactants and products. The hydraulic field includes two-step Darcy’s law. In MICP grouting repair, the multi-step grouting method is usually adopted, that is, the first step is to inject bacteria solution, and the second step is to inject cementation solution (urea solution with equal molar concentration and calcium source solution), in order to avoid the premature generation of calcium carbonate precipitation, reduce the plugging of the grouting port and the uneven distribution of calcium carbonate precipitation.

In this study, A numerical simulation method is employed to investigate the permeability reduction in sandy soil through MICP technology. After introducing a degree of model simplification, the subsequent analyses were undertaken as follows: (1) Convective diffusion as well as spatial and temporal distribution of the bacterial solution in porous media after injection. (2) Adsorption and recession of bacteria during the MICP reaction. (3) Spatial and temporal distribution of calcium carbonate precipitation from bacterial mineralization. (4) The effect of calcium carbonate precipitation on the reduction of permeability and porosity of sandy soils. (5) Adding boundary conditions and varying the grouting time, concentration, rate and number of times for variable parameter analysis to investigate the effect on the restoration effect.

### 2.2 Materials

**FIGURE 1:**
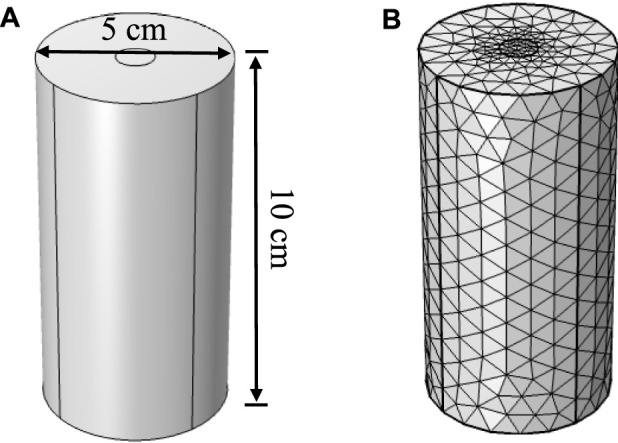
**(A)** Geometric model. **(B)** Mesh partition.

The model is designed to simulate the grouting test of a cylindrical sand column. The dimensions of the sand column are 5 cm in diameter and 10 cm in height. A grouting hole of 1 cm in diameter is set at the top of the sand column, with an outlet at the bottom. Bacterial solution and binding fluid are injected separately and allowed to naturally permeate and diffuse within the sand, ultimately reacting to form CaCO_3_ crystals, thereby achieving the reinforcement of the sand. The geometric structure of the model is shown in Figure 1A. Additionally, triangular mesh elements are chosen for discretization. The mesh division is illustrated in Figure 1B, and the information of the elements parameters by meshing is listed in Table 1.

**TABLE 1:** Element parameter.

Element size/m		Maximum element growth rate	Curvature factor	Narrow area resolution
Max	Min			
0.008	0.001	1.45	0.5	0.6

Element size, the length or width of element; Maximum element growth rate, determining the maximum rate of increase with element from small to large; Curvature Factor, determining boundary element size; Narrow Area Resolution, controlling the number of layers of element in narrower areas.

### 2.3 Parameters

The basic parameters of the model are shown in Table 2, and some of the parameters were selected based on referencing previous researchers’ studies, in conjunction with the actual circumstances of this simulation. The grouting scheme is presented in Table 3, while the boundary conditions are outlined in Table 4.

**TABLE 2:** Values of parameter used in the model.

Parameter	Symbol	Value	Unit	References
Density of water	*ρ*_*w*_	1,000	kg/m^3^	-
Viscosity of fluid	μ	0.001	Pa·s	-
Half-saturation constanty	*k* ^*m*^	55	mol/m^3^	Van Paassen (2009)
Molecular mass of urea	*m_urea*	0.078	kg/mol	-
Molecular mass of Ca^2+^	*m_Ca* ^*2+*^	0.04	kg/mol	-
Molecular mass of NH_4_^+^	*m_NH*_*4*_^*+*^	0.018	kg/mol	-
Molecular mass of CaCO_3_	*m*_*CaCO3*_	0.1	kg/mol	-
Density of CaCO_3_	*ρ*_*CaCO3*_	2,710	kg/m^3^	-
Diffusion coefficient	*D*_*m*_	2 × 10^−9^	m^2^/s	Wang and Nackenhorst (2020)
Maximum urease constant	*u*_*_sp*_	1.4 × 10^−8^	mol/m^3^/s	-
Time constant	*t*_*d*_	288,000	s	Fauriel and Laloui (2012)
Attachment rate	*k*_*att*_	1.52 × 10^−3^	1/s	-
Decay rate	*k*_*d*_	3.18 × 10^−7^	1/s	Wang and Nackenhorst (2020)
Permeability	*k*_*0*_	2 × 10^−12^	m^2^	Fauriel and Laloui (2012)
Porosity	*n*_*0*_	0.40	1	Fauriel and Laloui (2012)
Initial cell concentration	*C*0_*_*_ *bact*	7.2×10^5^	1	-

**TABLE 3:** Numerical simulation grouting scheme.

Group	Bacterial solution		Cementation solution			Grouting time
	Concentration	Retention time	Flow rate	Concentration	Retention time	
	(Cells/mL)	(h)	(m/s)	(mol/m^3^)	(h)	
S-7.2-3-1.5	7.2e5	1	1.5e-3	300	10	1
S-7.2-5-1.5	7.2e5	1	1.5e-3	500	10	1
S-7.2-10-1.0	7.2e5	1	1.0e-3	1,000	10	1
S-7.2-10-1.5	7.2e5	1	1.5e-3	1,000	10	1
S-7.2-10-2.0	7.2e5	1	2.0e-3	1,000	10	1
S-7.2-15-1.5	7.2e5	1	1.5e-3	1,500	10	1
S-4.3-10-1.5	4.3e5	1	1.5e-3	1,000	10	1
M-4.3-10-1.5	4.3e5	1	1.5e-3	1,000	10	5
M-7.2-10-1.5	7.2e5	1	1.5e-3	1,000	10	5
M-7.2-10-1.0	7.2e5	1	1.0e-3	1,000	10	5
M-7.2-10-2.0	7.2e5	1	2.0e-3	1,000	10	5
M-7.2-3-1.5	7.2e5	1	1.5e-3	300	10	5
M-7.2-5-1.5	7.2e5	1	1.5e-3	500	10	5
M-7.2-15-1.5	7.2e5	1	1.5e-3	1,500	10	5

**TABLE 4:** Setting of boundary conditions for numerical simulation.

Stage	Bacterial solution	Retention	Cementation solution
Upper boundary of sand column			
*q* ^*w*^	$q_{in1}^{w}$		$q_{in2}^{w}$
*P*_*l*_		*P*_*atm*_	
*C* ^*bacl*^	*C*0_*_*_ *bact*	$\left(D\cdot n\nabla C\right)\cdot n=0$	$\left(D\cdot n\nabla C\right)\cdot n=0$
*C* ^*urea*^	$\left(D\cdot n\nabla C\right)\cdot n=0$	$\left(D\cdot n\nabla C\right)\cdot n=0$	$C_{0}^{urea}$
$C^{{Ca}^{2+}}$	$\left(D\cdot n\nabla C\right)\cdot n=0$	$\left(D\cdot n\nabla C\right)\cdot n=0$	$C_{0}^{{Ca}^{2+}}$
$C^{{{NH}_{4}}^{+}}$	$\left(D\cdot n\nabla C\right)\cdot n=0$	$\left(D\cdot n\nabla C\right)\cdot n=0$	$C_{0}^{{NH}_{4}^{+}}$
Lower boundary of sand column			
*q* ^*w*^			
*P*_*l*_	*P*_*atm*_		*P*_*atm*_
*C* ^*bacl*^	$\left(D\cdot n\nabla C\right)\cdot n=0$	$\left(D\cdot n\nabla C\right)\cdot n=0$	$\left(D\cdot n\nabla C\right)\cdot n=0$
*C* ^*urea*^	$\left(D\cdot n\nabla C\right)\cdot n=0$	$\left(D\cdot n\nabla C\right)\cdot n=0$	$\left(D\cdot n\nabla C\right)\cdot n=0$
$C^{{Ca}^{2+}}$	$\left(D\cdot n\nabla C\right)\cdot n=0$	$\left(D\cdot n\nabla C\right)\cdot n=0$	$\left(D\cdot n\nabla C\right)\cdot n=0$
$C^{{{NH}_{4}}^{+}}$	$\left(D\cdot n\nabla C\right)\cdot n=0$	$\left(D\cdot n\nabla C\right)\cdot n=0$	$\left(D\cdot n\nabla C\right)\cdot n=0$

### 2.4 Model establishment and validation

#### 2.4.1 Bacterial behavior

The decay and attachment rates of bacteria are defined, while the growth and proliferation are not considered in this research. This omission is based on practical considerations within the Microbially Induced Calcite Precipitation (MICP) applications, where the bacterial solution is initially cultured and activated in the laboratory to meet the desired concentration and vitality prior to its use in experiments. The numerical model posits that the decline in bacterial populations is predominantly determined by the decay rate (*k*_*d*_), which is presumed to have a linear relationship with the decay process. The model further assumes a uniform decay rate for both attached and suspended bacteria. As a result, the temporal variation in the total bacterial concentration is depicted by the following relationship:

$$\frac{\partial C^{bact}}{\partial t}=‐k_{d}C^{bact}$$ (4)

In the paper, the bacteria solution is present in two forms, as suspended bacteria that move with the medium solution, denoted as (*C* ^*bacl*^), and as bacteria attached to solid particles, which remain immobile, represented by (*C* ^*bacs*^). The quantity of attached bacteria is contingent upon the number of suspended bacteria in the environment. Previous studies have indicated that bacterial attachment can be defined by a first-order kinetic model with a constant attachment rate (*k*_*att*_).

Attached bacteria are defined by the following equation:

$$\frac{\partial C^{bacs}}{\partial t}=k_{att}C^{bacl}‐k_{d}C^{bacl}$$ (5)

Suspended bacteria are defined by the following equation:

$$\frac{\partial C^{bacl}}{\partial t}={‐k}_{att}C^{bacl}‐k_{d}C^{bacs}$$ (6)

The reactive transport equations for bacteria solution can be explicitly expressed as:

$$n\frac{\partial C^{bacl}}{\partial t}=\nabla \left(nD^{bacl}\cdot \nabla C^{bacl}\right)‐u\cdot \nabla C^{bacl}‐nk_{d}C^{bacl}‐nk_{att}C^{bacl}$$ (7)

#### 2.4.2 Overall kinetically controlled reaction model

Similar to the research done by Fauriel and Laloui. (2012) and Van Wijngaarden et al. (2011), a global kinetic control reaction model was used to describe the urea hydrolysis and calcite precipitation in MICP. The chemical reaction is assumed to be a first-order reversible reaction and controlled by the total kinetic rate krea (*k_urea*). Based on the phenomenon that urease activity will decline with time (Van Paassen, 2009), this paper uses an exponential equation to define the change of urease activity with time *f*_d_ (t) = exp [- *k*_*d*_ (*t-t*_*c*_)], *t*_*c*_ is the grouting time of cementation solution, s. Combined with Michaelis-Menten kinetics equation, the urea hydrolysis rate equation can be obtained as follows:

$$k_{\_urea}=U_{\max}\frac{C^{urea}}{k^{m}+C^{urea}}\exp\left(‐\frac{t}{t_{d}}\right)$$ (8)

Where: *U*_max_ is the maximum hydrolysis rate of urea, mol/(m^3^/s); *k*_*m*_ is the half-saturation constant, mol/m^3^; *C*_*urea*_ is the concentration of urea, mol/m^3^; *t*_*d*_ is the time constant, s.

$$U_{\max}=u_{\_\text{sp}\cdot }C^{\text{bact}}$$ (9)

Where: *u_*_*sp*_ is the maximum urease constant; *C*_*bact*_ is the total concentration of bacterial solution.

#### 2.4.3 Mass balance equation of liquid phase

There are five components in the liquid phase: pore water, calcium acetate, urea, ammonium and suspended bacteria. Because the dynamic viscosity of urea solution and calcium acetate solution is low, the dynamic viscosity and density of bacterial solution can be considered to be the same as that of aqueous solution, and the flow of solution in porous media conforms to Newtonian fluid law. Therefore, the grouting process of cementation solution and bacterial solution in porous media can be defined by Darcy’s law physical field equation.

$$\frac{\partial n}{\partial t}=‐\nabla \cdot u$$ (10)

where: *n* is the porosity; *t* is time, s; *u* is the vector of liquid velocity of the Darcy field, m/s.

The percolation rate of pore water can be defined by the following equation:

$$u=‐\frac{k}{\mu }\left(\nabla P+\rho gz\right)$$ (11)

Where: *k* is the permeability coefficient of the porous medium, m^-2^; *μ* is the dynamic viscosity, Pas; *P* is the liquid pressure in the porous medium; *ρ* is the density of the fluid, kg/m^3^; *g* is the gravity acceleration vector; *z* is the vertical coordinate in the three-dimensional model space.

The macroscopic mass balance was applied to derive the mathematical model. Since the liquid phase contains five components, the general expression of the liquid phase mass balance equation is as follows:

$$\frac{\partial \left(n\rho_{l}\right)}{\partial t}=‐\nabla \cdot \left(n\rho_{l}u\right)+\Omega^{urea}+\Omega^{{Ca}^{2+}}+\Omega^{{{NH}_{4}}^{+}}+Q^{w}$$ (12)

Where: *n* is the porosity; *u* is the vector of liquid velocity; *Ω* ^*i*^ is the production/consumption rate of each aqueous chemical mass; *Q* ^*w*^ is the source term of pore water mass; *ρ*_*l*_ is the liquid density.

According to the Van Wijngaarden et al. (2011) experiment, a linear relationship was found between the density of the liquid and the concentration of each substance:

$$\rho_{l}=\rho_{w}+0.0154994\text{kg}/\text{mol}C^{urea}+0.0867338\text{kg}/\text{mol }C^{Ca^{2+}}+0.0158991\text{kg}/\text{mol }C^{{NH}_{4}^{+}}$$ (13)

Where: *ρ*_*w*_ is the constant water density; *C* ^*i*^ is the concentration of urea, calcium, and ammonium in solution.

It is assumed that the advective transport of all components in the liquid phase is controlled by the same flow rate. According to the mass balance equations for each species, the transport equations for urea, calcium, ammonium and suspended bacteria can be expressed as:

$$n\frac{\partial C^{i}}{\partial t}=\nabla \cdot \left(nD_{i,j}\cdot \nabla C^{i}\right)‐q\nabla C^{i}+q^{i}$$ (14)

$$D_{i,j}=\left(\alpha_{L}‐\alpha_{T}\right)\frac{v_{i}⊗v_{j}}{\left|v\right|}+D_{m}$$ (15)

Where: *C* ^*i*^ is the concentration of each components, *i*∈(Urea, Ca^2+^, NH_4_^+^); *q* ^*i*^ is the reaction-related source term, *q = nu*; *D* is the diffusion-dispersion tensor: *α*_*L*_ is the longitudinal dispersion coefficient; *α*_*T*_ is the transverse dispersion coafficient; *v*_*i*_ = *u*_*i*_/*n* is the pore flow rate; *D*_*m*_ is the diffusion coefficient.

#### 2.4.4 Effect of calcium carbonate on porosity and permeability

In the system, once the produced calcium carbonate reaches a state of supersaturation, the precipitation of calcium carbonate crystal begins, serving to fill voids and act as a binder, thereby reducing porosity and permeability. This paper does not take into account the transformation of calcium carbonate crystal morphology, assuming that the precipitated calcium carbonate remains stationary. Consequently, the partial differential equation describing its concentration does not include terms for advection or diffusion. The equation governing the temporal change in calcium carbonate concentration is as follows:

$$\frac{\partial C^{{CaCO}_{3}}}{\partial t}=m_{{CaCO}_{3}}nk_{\_urea}$$ (16)

The equation of porosity change with time is as follows:

$$\frac{\partial n}{\partial t}=‐\frac{1}{\rho_{{CaCO}_{3}}}\frac{\partial C^{{CaCO}_{3}}}{\partial t}$$ (17)

Combining Eqs 16, 17, the porosity reduction equation of porous media caused by calcium carbonate precipitation can be obtained:

$$\frac{\partial n}{\partial t}=‐\frac{m_{{CaCO}_{3}}}{\rho_{{CaCO}_{3}}}nk_{\_urea}$$ (18)

*Kozeny-Carman* equation can be used to determine the inherent permeability. This equation is a commonly used formula for determining permeability and porosity in groundwater flow model. Combined with *K-C* equation, the permeability control equation can be obtained as follows:

$$k=\frac{{\left(d_{m}\right)}^{2}}{180}\frac{n^{3}}{{\left(1‐n\right)}^{2}}$$ (19)

Where: *C*_*CaCO3*_ is the concentration of precipitated calcium carbonate crystals; *m*_*CaCO3*_ is the molar mass of calcium carbonate; *ρ*_*CaCO3*_ is the density of calcium carbonate; *k* is permeability; *d*_*m*_ is the average particle size of the filling material.

#### 2.4.5 Model validation

**FIGURE 2:**
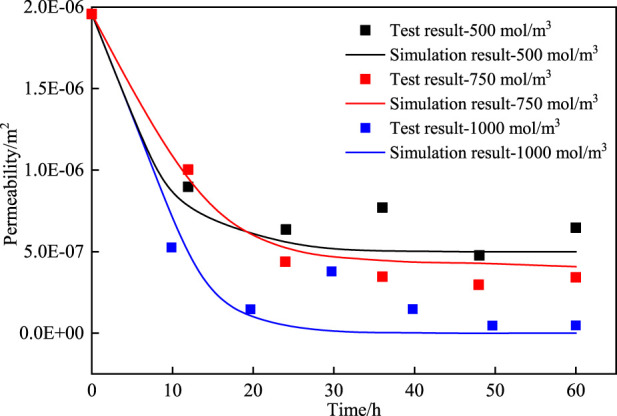
Comparison of numerical simulation results with experimental.

To validate the theoretical framework of model construction and the appropriateness of parameter selection, a validation model was established based on the initial conditions and methodologies used in existing experiment (Li, 2022), and the results were compared with the experimental data. The permeability at three cementation solution concentrations (500 mol/m^3^, 750 mol/m^3^, 1,000 mol/m^3^) were calculated following the existing test (Li, 2022). Figure 2 shows the comparison curve of permeability between experimental and theoretical values, along with the calculated correlation. The findings reveal that the calculated results generally agree with the measured results, and the two results are in a strong agreement. The feasibility of the numerical method was confirmed.

## 3 Results and discussion

### 3.1 Effect of initial bacteria solution concentration

**FIGURE 3:**
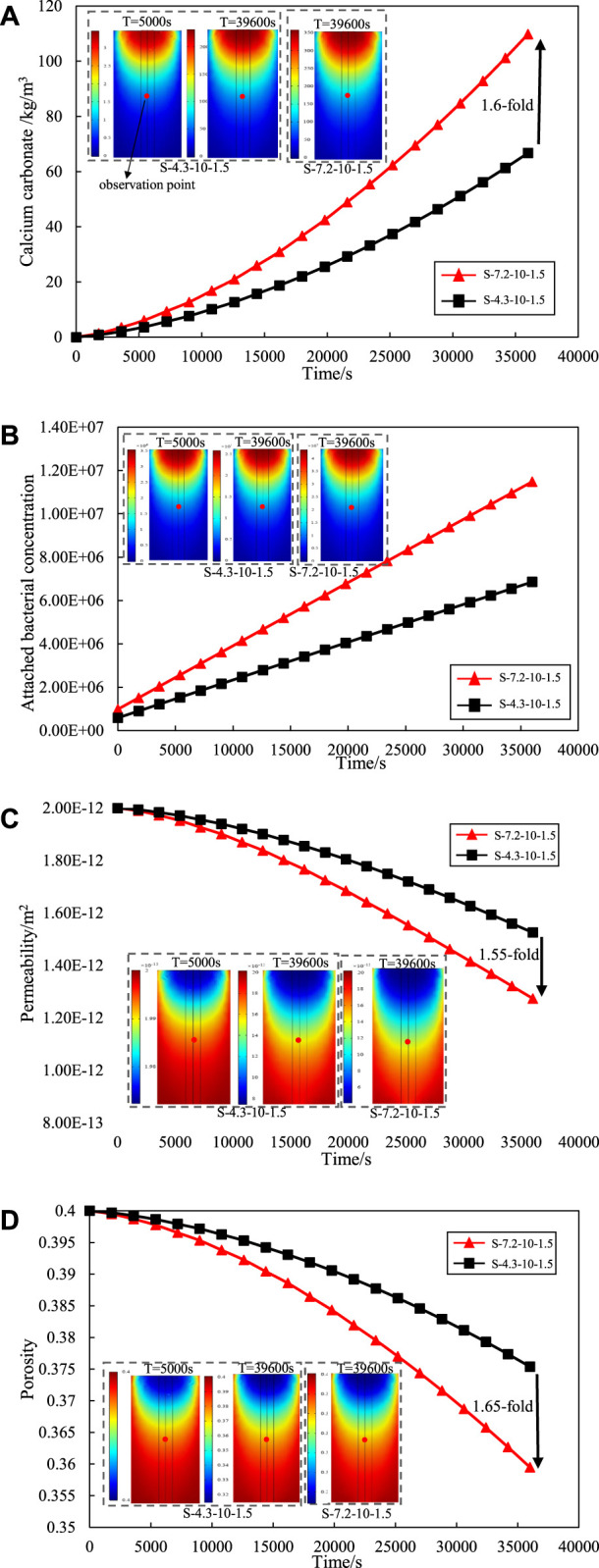
Effect of initial concentration of the bacterial solution on **(A)** calcium carbonate production. **(B)** attached bacterial. **(C)** permeability. **(D)** porosity.

Figure 3A illustrates the dynamic process of calcium carbonate production during grouting at the observation point (the center of the sand column) under the conditions of two different initial bacterial solution concentrations. The initial concentration of bacteria was determined by the cultivation time of bacteria. The amount of calcium carbonate increased with the reaction time, as shown as Figure 3A. Moreover, the calcium carbonate generation was increased by about 1.6-fold at the end of grouting following increase of the initial microbial concentration from 4.3E5 cells/mL to 7.2E5 cells/mL. Therefore, the amount of calcium carbonate is determined by the initial concentration of the bacterial solution at the same conditions. The variation in the results of calcium carbonate production also can be explained by the variation curve of the number of the attached bacterial concentration shown in Figure 3B. The number of suspended bacteria transformed to the adsorbed state during the grouting process increased with higher the initial concentration of bacteria. Under the same conditions, more attached bacteria are involved in the MICP reaction and result in more calcium carbonate generation.

Figure 3C shows the variation curve of the permeability at the observation point of the sand column by the initial concentration of the bacterial solution. The trend of bacterial solution concentration on sand column permeability is consistent with the trend of calcium carbonate production. With increasing bacterial concentration, more attached bacteria participate in the reaction and generate more calcium carbonate precipitation. This precipitation exerts a sealing effect on the interstitial space between sand particles, leading to a reduction in the porosity of the sand column. The variation curve of the porosity at the observation point of the sand column with the concentration of bacterial solution is shown in Figure 3D. With the initial bacterial solution concentration of 4.3E5 cells/mL, the permeability and porosity at the end of grouting are reduced by 23.50% and 6.15% respectively; With the initial bacterial solution concentration of 7.2E5 cells/mL, the permeability at the end of grouting decreases by 36.50% and 10.13%. At the end of grouting, the initial bacterial concentration of 7.2E5 cells/mL increased the repair effect of permeability by 1.55-fold and the repair effect of porosity by 1.65-fold compared to 4.3E5 cells/mL. This underscores the pivotal role of the initial bacterial concentration in influencing the grouting effect.

**FIGURE 4:**
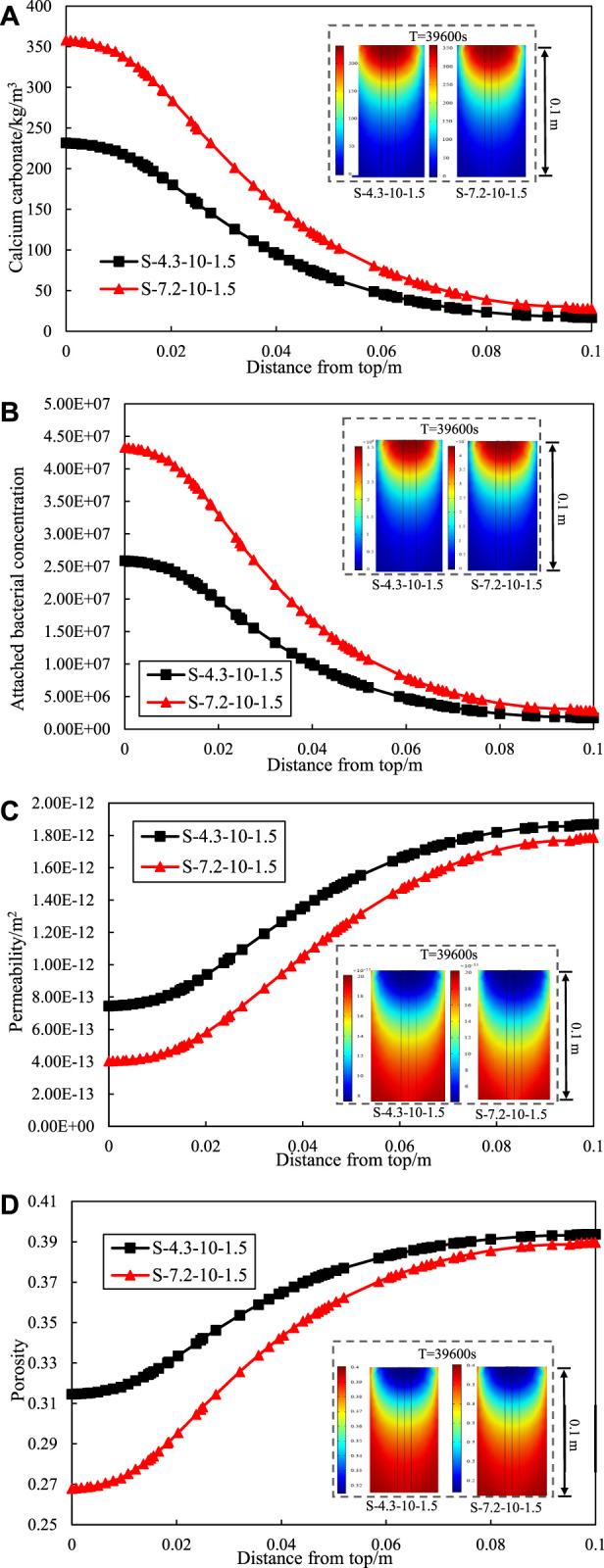
Effect of initial concentration of the bacterial solution on **(A)** calcium carbonate production. **(B)** attached bacterial. **(C)** permeability. **(D)** porosity of different depths.

Figure 4A shows the variation of calcium carbonate production with height of the sand column. It can be seen that the calcium carbonate production is highest at the entrance (top of the sand column) and lowest at the exit (bottom of the sand column). The phenomenon is due to priority reaction of sand near the grouting entrance with the bacterial solution, which results in the production of a higher amount of calcium carbonate. The produced calcium carbonate fill the void in the sand column and prevent the bacterial solution and cementation concentration from spreading downward. It leads to a low calcium carbonate production at the bottom of the sand column. Meanwhile, the variation curve of Attached bacterial concentration along the depth further explains the above phenomenon, as shown as Figure 4B.

Moreover, the trend of calcium carbonate production along the depth at different initial bacteria solution concentration remained consistent. The calcium carbonate production at the entrance increased with higher initial bacterial solution concentration, while the calcium carbonate production at the exist varied insignificantly. It means that increasing the initial bacterial solution concentration increases the inhomogeneity of calcium carbonate distribution in the sand column with depth. Meanwhile, grouting from one side lead to an uneven distribution of calcium carbonate production in the sand column along the depth. The phenomenon of clogging seems to occur in the half-way of the 100 mm long sample. However, it is hard to reinforce the soil foundation by grouting from both ends in practice. Therefore, Omoregie et al. (2019) proposed the use of single-phase low pH injection and low temperature to alleviate the problem of uneven calcium carbonate generation.

Figures 4C, D respectively show the variation of permeability and porosity with height of the sand column. The reaction of the bacteria solution concentration and cementation solution concentration around the treatment point of entrance limits penetration into the sand. At the entrance, the increasing of calcium carbonate production significantly reduced the permeability and porosity of the sand column. It leads to less calcium carbonate formation inside the soil matrix and uneven calcium carbonate distribution.

### 3.2 Effect of cementation solution concentration

**FIGURE 5:**
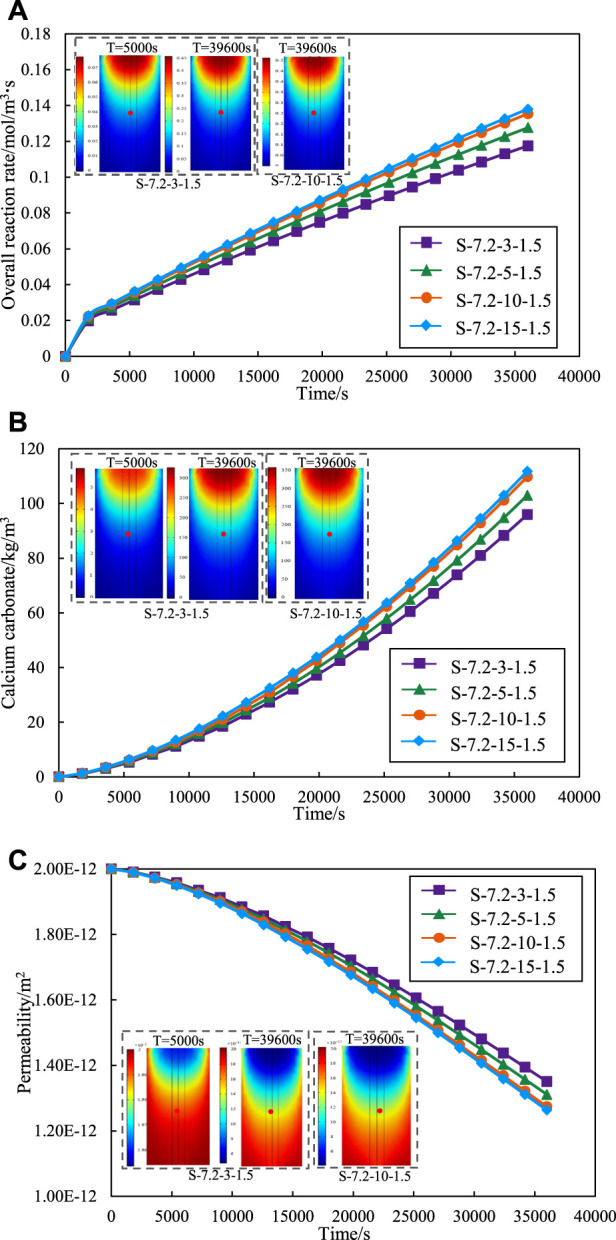
Effect of different cementation solution concentration on **(A)** overall reaction rate. **(B)** calcium carbonate production. **(C)** permeability.

Figure 5A summarizes the variation curves of the effect of different concentrations of cementation solution on the overall reaction rate of MICP. The overall reaction rate also increases when the concentration of the cementation solution increases from 300 to 1,500 mol/m^3^. However, the intervals between each curve is diminish progressively, which indicates that there is no nonlinear increase between the reaction rate and the concentration of the cementation solution, and the promotion of the cementation solution becomes weaker. When the concentration of the cementation solution is increased from 1,000 to 1,500 mol/m^3^, the reaction rate is almost the same, and there is no significant increase.

Figure 5B shows the impact of varying cementation solution concentration on calcium carbonate production. The graph reveals a concurrent increase in the quantity of calcium carbonate with the elevation of cementation solution concentration. A notable observation is that the quantity of calcium carbonate remains nearly constant as the concentration of the cementation solution escalates from 1,000 to 1,500 mol/m^3^. It is similar to the regularity exhibited in Figure 5A. A heightened reaction rate correlates with an increased production of calcium carbonate within the same time. The reaction rates for concentrations of 1,000 and 1,500 mol/m^3^ are nearly identical, resulting in a comparable amount of calcium carbonate generated at both concentrations within the same timeframe.

Figure 5C shows the variation of permeability of various sand columns during grouting with different cementation solution concentration. The increase of concentration will make the permeability decrease more, but the promotion effect on the permeability decrease will be smaller and smaller. This is because the decrease of permeability is caused by the formation of calcium carbonate precipitation. Therefore, the permeability curve shows the same change trend as the amount of calcium carbonate.

### 3.3 Effect of grouting rate

**FIGURE 6:**
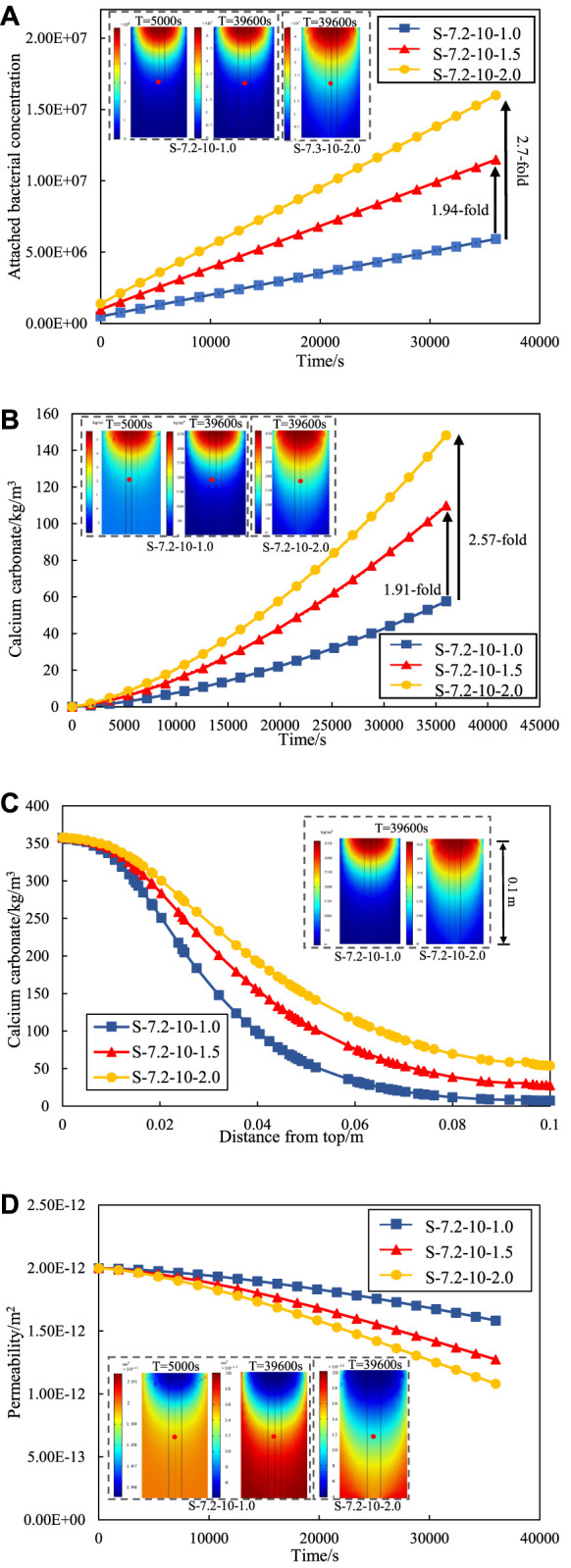
**(A)** Number of attached bacterial concentration under different grouting rates. **(B)** Calcium carbonate precipitation under different grouting rates. **(C)** Calcium carbonate precipitation along the depth at different grouting rates **(D)** Permeability change curve under different grouting rates.

Figure 6A shows the variation curve of attached bacteria at the observation point under different grouting rates. The number of attached bacteria increases rapidly with the grouting proceeded at the same grouting rate. This phenomenon is attributed to the gradual attachment and fixation of suspended bacteria (initially injected bacteria) on the surface of sand particles, leading to converted into attached bacteria. Simultaneously, an increase in the grouting rate correlates with a heightened abundance of attached bacteria. At the end of grouting, the attached bacteria at the grouting rate of *v* = 1.5E-3 m/s is 1.94-fold that of *v* = 1.0E-3 m/s, and the attached bacteria at the grouting rate of v = 2.0E-3 m/s is 2.70-fold that of *v* = 1.0E-3 m/s.

The grouting method of the numerical model in this paper follows stepwise approach. The bacterial solution is injected first and then cementation solution. Figure 6B shows the amount of calcium carbonate generated during the grouting process at the observation point at different grouting rates. The study is concerned with the amount of calcium carbonate generated during the grouting process. An assumption is made that the calcium carbonate precipitation remains stationary, thereby excluding considerations of erosion caused by high-speed grouting. Consequently, a amount of generated calcium carbonate will exhibit a continual rise throughout the grouting process. The calcium carbonate concentration increases with the increase of the grouting rate. At the end of grouting, the calcium carbonate generated by the grouting rate of *v* = 1.5E-3 m/s is 1.91-fold of the grouting rate of *v* = 1.0E-3 m/s, and the calcium carbonate generated by the grouting rate of *v* = 2.0E-3 m/s is 2.57-fold of the grouting rate of *v* = 1.0E-3 m/s. The variation curve of calcium carbonate production along the depth at different grouting rates are shown in Figure 6C. The calcium carbonate production at the entrance is almost the same due to the same bacterial solution concentration and cementation solution concentration. However, the calcium carbonate production at the exit of the sand column at different grouting rate has significant variations. The lowest calcium carbonate production was found at the exit by the grouting rate of v = 1E-3 m/s, and the highest calcium carbonate production was found by the grouting rate of v = 2E-3 m/s. The indicates that increasing the grouting rate can alleviate the problem of uneven distribution of calcium carbonate with depth in the sand column to some extent. The rationale behind this phenomenon lies in the adoption of a higher grouting rate, resulting in enhanced transport efficiency of Microbially Induced calcite Precipitation (MICP) reaction material. The elevated efficiency, coupled with an escalated reaction rate, facilitates earlier and faster contact between the reactant and the bacteria preemptively injected into the sand column.

Consequently, the hydrolysis reaction of urea transpires more promptly, leading to an increased generation of calcium carbonate precipitates. Conversely, a reduction in the grouting rate leads to a diminished quantity of calcium carbonate. The occurrence is attributed to the decreased grouting rate, resulting in an extended grouting time. The prolonged duration delays the time for cementation to establish full contact and reaction with bacteria. Consequently, bacterial activity is attenuated, ultimately resulting in a decreased production of calcium carbonate.

Figure 6D illustrates the variation of permeability of observation points under different grouting rates. The permeability of the sand column decrease with time and rate of grouting as grouting progresses. The reason for the decrease of permeability is that the calcium carbonate precipitation generation fills the voids between the sand particles in the MICP process. According to the comparison data, the permeability decreased by 21.0% with the grouting rate of *v* = 1.0E-3 m/s; With the grouting rate of *v* = 1.5E-3 m/s, the permeability decreased by 36.0%; With the grouting rate of *v* = 2.0E-3 m/s, the permeability decreased by 46.0%. Meanwhile, existing studies also have shown that the grouting rate has a great effect on the amount of calcium carbonate generation. Wu et al. (2019) found that a higher grouting rate would increase the formation rate of calcium carbonate when grouting rock fractures. Therefore, the effect of MICP repair be controlled by adjusting the grouting rate, and the repair effect be further improved by means of low rate grouting at the initial stage and high rate grouting at the later stage.

### 3.4 Effect of grouting times

**FIGURE 7:**
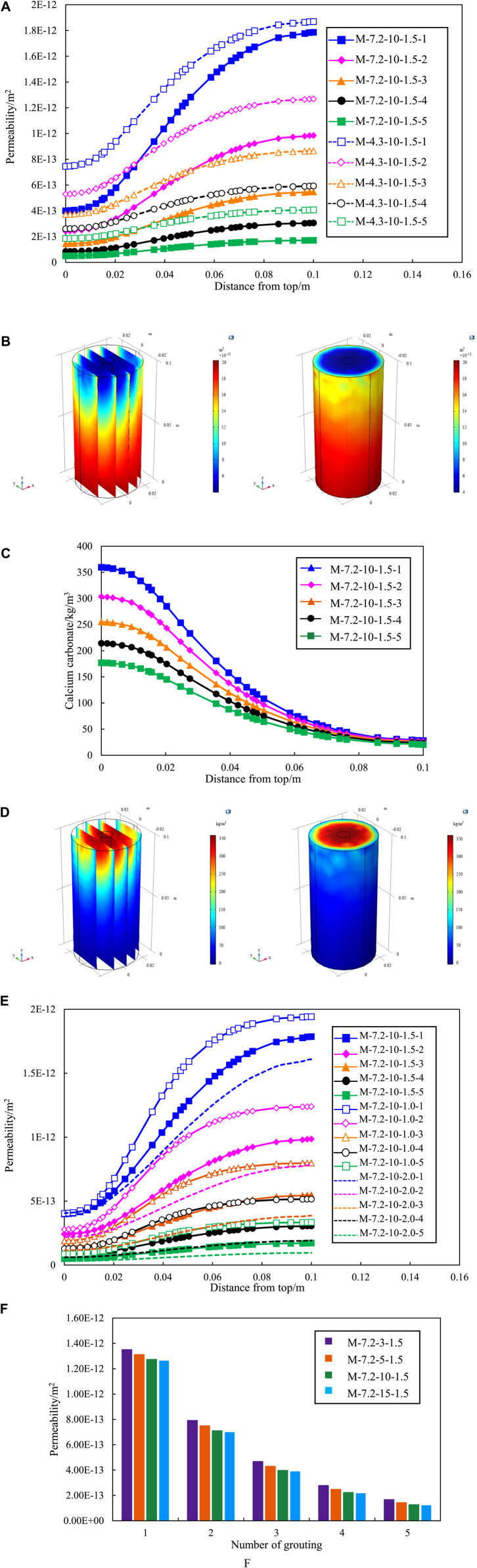
(Continued).

The efficacy of single round grouting frequently falls short in achieving optimal repair effect. The repair effect of multiple grouting is investigated to solve the problem of single round grouting. Each round of grouting lasts for 10 h and the maximum number of grouting in this simulation is 5 times. Figure 7A shows the impact of multiple rounds of grouting on permeability under group M-7.2-10-1.5 and M-4.3-10-1.5. The permeability of the sand column is significantly reduced by multiple grouting and. In Group M-7.2-10-1.5, the permeability at the grout hole location was reduced from 4.57E-13 m^2^ to 5.08E-14 m^2^ over the first round to the fifth round of grouting, resulting in an 11.12-fold reduction in sand column permeability. From Figure 7A it can be found that the remediation efficacy of bacteria with a concentration of 7.2E5 cells/mL after two grouting sessions is approximately equivalent to that of bacteria with a concentration of 4.3E5 cells/mL after three grouting sessions. The permeability of high concentration bacteria after four grouting sessions exhibits a 24.96% reduction compared to low concentration bacteria after five grouting sessions. Hence, during the execution of practical engineering grouting repairs, careful consideration should be given to the bacterial culture duration and the choice of bacterial solution with elevated culture concentration.

Figure 7A also shows the variation of permeability with sand column height. It can be seen that the trend remains consistent under different bacterial concentration. Taking group M-7.2-10-1.5-4 as an example, the lowest permeability is at the entrance (top of the sand column) and the highest is at the exit (bottom of the sand column), as shown as Figure 7B. The reason for this phenomenon can be explained by the variation of calcium carbonate content with sand column height shown in Figure 7C. The concentration of substances near the grouting mouth is elevated, gradually diminishing towards the outer boundary of the model. The components persistently diffuse outward, leading to concentration increasing and eventual migration of diffusion to the exit position, as shown as Figure 7D. The decrease of permeability of sand column is mainly controlled by calcium carbonate produced by microorganism. The higher the amount of calcium carbonate produced, the more the permeability decreases.

Simultaneously, Figure 7A shows hat with the increase of grouting times, the interval between the curves becomes smaller, showing a rule from thin to dense. The observation suggests that, with the escalating number of grouting sessions, the efficacy of single round grouting diminishes and the production of calcium carbonate per grouting session decreases. The decline in permeability is directly influenced by the quantity of calcium carbonate, as shown as Figure 7C. Hence, the reduction in the quantity of calcium carbonate produced per grouting session leads to a decline in the standalone reparative effect of each grouting round.

Figure 7E summarizes the impact of multiple rounds of grouting on the permeability of the sand column under three distinct grouting rate scenarios. Permeability was significantly different at varying grouting rates for the same grouting sessions. The reparative efficacy of two grouting sessions at a rate of v = 2.0E-3 m/s surpasses that of three sessions at a rate of v = 1.0E-3 m/s. The repair effect of three grouting sessions at a rate of v = 1.5E-3 m/s closely parallel those of four sessions at a rate of v = 1.0E-3 m/s. The reparative effectiveness of four grouting sessions at a rate of v = 2.0E-3 m/s is nearly equivalent to that of five sessions at a rate of v = 1.5E-3 m/s, and markedly superior to the outcomes of five sessions at a rate of v = 1.0E-3 m/s.

The effect of multiple rounds of grouting on the permeability of sand column under four different concentrations of cementation solution, as shown as Figure 7F. With an equivalent number of grouting sessions, the disparity in permeability under varying concentrations of cementation solution is not substantial. The influence of cementation solution concentration on permeability is considerably less pronounced compared to the effect of increasing grouting sessions. Therefore, to optimize cost-effectiveness, a prudent choice for cementation solution concentration during MICP grouting repair would be 500 mol/m^3^.

**FIGURE 8:**
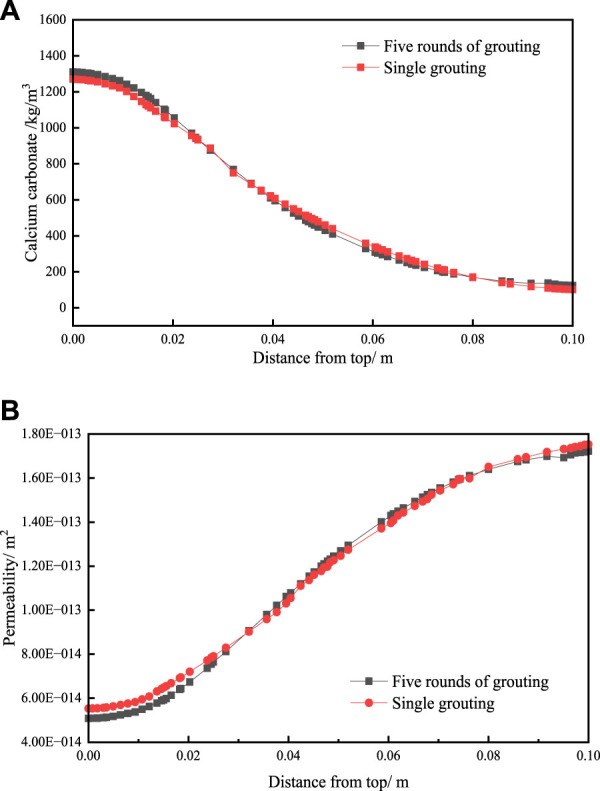
The variation of **(A)** calcium carbonate generation and **(B)** permeability after five rounds of grouting and single grouting.

The variation of calcium carbonate generation and permeability after five rounds of grouting and single grouting with a single separate injection of the same volume of bacterial solution and cementing solution, as shown as Figure 8. The propagation of bacteria during the grouting process is not considered in this paper, while no time interval is set between multiple rounds of grouting process. So, the variation of calcium carbonate production and bacteria concentration is continuous in each round during multiple rounds of grouting. The results of multiple grouting with a single separate injection of the same volume of bacterial solution and cementing solution have not significant differences.

## 4 Conclusion

The paper investigated the influence of initial bacterial solution concentration, cement concentration, grouting rate, and grouting time on bacterial adsorption, calcium carbonate precipitation, and permeability in sand columns. The key conclusions can be summarized as follows:

(1) A numerical simulation method was established in this paper to investigate the factors affecting the microbial grouting process in sand columns. The numerical method was validated by comparing its predictions with experimental results available in the literature.
(2) The increasing initial bacterial solution and cementation solution concentration significantly improved the calcium carbonate production. The continuous generation of calcium carbonate clogged the void of the sand column, which caused the permeability and porosity to decrease and prevent the bacterial solution and cementation concentration from spreading downward. It leads to an uneven distribution of calcium carbonate in the sand column.
(3) The grouting rate had a significant effect on the amount of calcium carbonate generated. The grouting rate increased with the increase of the transportation efficiency of the bacterial solution. Based on this, the problem of uneven distribution of calcium carbonate with depth in the sand column was alleviated, and the permeability of the sand column was effectively reduced.
(4) Multiple grouting improved the MICP repair effect significantly, compared with the first grouting. The calcium carbonate productions increased with the times of grouting. Permeability and the efficacy of grouting decreases with the times of grouting.

## Data Availability

The original contributions presented in the study are included in the article/Supplementary material, further inquiries can be directed to the corresponding authors.
